# Characterization of the Gene *BmEm4*, a Homologue of *Drosophila E(spl)m4*, from the Silkworm, *Bombyx mori*

**DOI:** 10.1155/2009/947490

**Published:** 2009-10-12

**Authors:** Fenghui Zeng, Hongxia Xie, Zuoming Nie, Jian Chen, Zhengbing Lv, Jianqing Chen, Dan Wang, Lili Liu, Wei Yu, Qing Sheng, Xiangfu Wu, Yaozhou Zhang

**Affiliations:** ^1^Institute of Biochemistry, Zhejiang Sci-Tech University, Hangzhou 310018, China; ^2^Institute of Biochemistry, College of Life Sciences, Zhejiang Provincial Key Laboratory of Silkworm Bioreactor and Biomedicine, Hangzhou 310018, China; ^3^Xin Yuan Institute of Medicine and Biotechnology, Zhejiang Sci-Tech University, Hangzhou 310018, China

## Abstract

The *Drosophila E(spl)m4* gene contains some highly conserved motifs (such as the Brd box, GY box, K box, and CAAC motif) in its 3′ untranslated region (3′ UTR). It was shown to be a microRNA target gene in *Drosophila* and to play an important role in the regulation of neurogenesis. We identified a homologue of the *E(spl)m4* gene from *Bombyx mori* called *BmEm4* and examined the expression patterns of BmEm4 mRNA and protein. There was a lack of correlation in the expression of the mRNA and protein between the different developmental stages, which raises the possibility of posttranscriptional regulation of the BmEm4 mRNA. Consistent with this idea is the finding that the 3′ UTR contains two putative binding sites for microRNAs. Moreover, given that the expression is the highest in the larval head, as confirmed by immunohistochemistry, we propose that *BmEm4* may also be involved in the regulation of neurogenesis. Immunostaining indicated that BmEm4 is located primarily in the cytoplasm.

## 1. Introduction

The Notch signaling pathway plays a fundamental role during the specification of cell fates in the development of multicellular animals [1–3]. The pathway comprises four core elements: the Notch receptor, ligands of the DSL (Delta/Serrate/Lag-2) family, the transcriptional cofactor CSL (CBF1/Suppressor of hairless/Lag-1), and target genes such as *HES* (*Hairy/Enhancer of Split*) [4]. Direct binding of a ligand on the surface of the signaling cell to a Notch receptor in the plasma membrane of the target cell initiates two successive proteolytic cleavages by TACE (TNF-*α*-converting enzyme) and the *γ*-secretase/presenilin complex, respectively. These cleavage events result in the release of the Notch intracellular domain (NICD), which can then enter into the nucleus. In the nucleus, the NICD interacts directly with the DNA binding protein CSL and activates the transcription of target genes such as HES [5]. Genes of the *Enhancer of split* complex (E(spl)-C) are also targets of the Notch signaling pathway; they have been studied mainly in *Drosophila* [6, 7]. The complex harbors 12 transcription units: the E(spl) bHLH genes, *m* *β*, *m* *γ* *, m*δ*, m3, m5, m7, *and* m8*, are seven Notch-responsive basic helix-loop-helix (bHLH) transcription factors that act to repress neural cell fate [7–10]; whereas *E(spl)* *m* *α* *, m2, m4, *and* m6* are members of the *Bearded* (*Brd*) gene family, whose overexpression antagonizes Notch signaling activity [11–13]. The remaining gene *m1* appears unrelated to the Notch pathway and probably encodes a protease inhibitor of the Kazal family [12, 13].* E(spl) m4* is expressed specifically in the proneural clusters of the imaginal discs in *Drosophila* [6, 14]. Several E(spl)-C genes, including *m4*, are known to be activated directly by the transcription factor Suppressor of Hairless (SuH) in response to Notch receptor activity [6, 15, 16]. The *Brd* family genes also regulate neural development, via the formation of RNA:RNA duplexes with proneural transcripts [17]. They might be able to be substitute for each other, and this redundancy means that the expected phenotypes, most notably bristle loss, are not observed in *Brd* mutants [13, 18].

Two sequence motifs, named the Brd box (AGCTTTA) and GY box (GTCTTCC), were found first in the 3′ untranslated region (3′ UTR) of *Brd* and then in most genes of the E(spl)-C [17, 19]. Another two novel boxes have also been reported. The first is a hexamer motif named the K box (TGTGAT; rich in keto nucleotides), which is present in the 3′ UTRs of seven genes of the E(spl)-C (including *m4*) [20]. An additional sequence, the CAAC motif, which is associated closely with the Brd, GY, and K boxes in the 3′ UTR of E(spl)-C genes has been reported [12]. Cumulative evidence from a series of genetic and bioinformatic studies over the past decade suggests that the Notch signaling pathway is regulated by microRNAs (miRNAs) that target the GY-box, Brd-box, and K-box motifs in *Drosophila* [1, 3, 21–23]. Taken together, the *Brd* family genes are likely to be integral members of the Notch pathway and to play important roles as effectors or modulators of this pathway [24].

The silkworm, *Bombyx mori*, an insect that undergoes complete metamorphosis, not only has great agronomic value but is also an excellent model for the study of insect genetics and molecular biology. However, no studies on *Brd* family genes in silkworm or any other Lepidoptera have been reported, and no information on the expression or subcellular localization of the proteins encoded by the silkworm *Brd* family genes is currently available.

In our previous study, which combined a computational method based on sequence homology searches with experimental identification based on microarray assays and Northern blotting, we reported the identification of the first set of *B. mori* miRNA genes and their predicted target genes (which included *BmEm4*) from larva, moth, and pupa [25]. In the study reported herein, we cloned one of the target genes *BmEm4* (GenBank accession number FJ436408) from silkworm and found it to be an ortholog of the *E(spl) m4* gene from *D. melanogaster* by bioinformatic analysis of the sequence. We used purified recombinant BmEm4 to generate a polyclonal antibody and analyzed its expression pattern in different developmental stages and in tissues of fifth instar silkworm larvae. The subcellular localization was analyzed by confocal immunofluorescence microscopy in a *B. mori* cell line.

## 2. Materials and Methods

### 2.1. Materials

The *Escherichia coli* strains TG1 and BL21(DE3) were grown at 37°C in LB medium, pH 7.5 (5 g of yeast extract, 10 g of tryptone and 10 g of NaCl per liter). The *B. mori* strain used in this study was Qingsong × Haoyue. Silkworms were reared on mulberry leaves under standard conditions. Trachea, heads, fatty bodies, testes, guts, Malpighian tubules, epidermises, ovaries, and silk glands were dissected from fifth instar larvae, frozen immediately in liquid nitrogen, and stored at −80°C. Pupae, at three days after pupation; day-3 fifth instar larvae, that is, after removal of mulberry leaves from their guts; nascent eggs; and moths were also frozen in liquid nitrogen and stored at −80°C. 4-6-Diamidino-2-phenylindole (DAPI) was purchased from Sigma (USA). The silkworm ovary cell line Bm5 (a gift from Prof. Zhi-Fang Zhang) was cultured in TC-100 medium (Sigma) supplemented with 10% fetal calf serum (FCS; Gibco BRL, USA) at 27°C.

### 2.2. Sequence Analysis

Analysis of the similarity of nucleotide and protein sequences was performed using the BLAST algorithm from NCBI (http://www.ncbi.nlm.nih.gov/). The characteristics of the gene were analyzed using DNAstar software (DNASTAR, Inc., USA). The orthologous sequences used for multiple sequence alignments were obtained from NCBI. For sequence alignment, we used Clustal W and BioEdit version 7.01 (Ibis Biosciences, CA). The positions at which the degree of amino acid identity between orthologs was more than 60% were denoted by shadowing.

### 2.3. Cloning, Expression and Antibody Preparation of BmEm4 in Silkworm

Total RNA was extracted from silkworm pupae using Trizol reagent (Invitrogen, USA) according to the manufacturer's instructions. Contaminating genomic DNA (gDNA) was removed by treatment with DNase I (TaKaRa, Japan). The ratios of OD 260/280 for the RNA samples were between 1.8 and 2.1, as determined by UV spectrophotometry (NanoDrop ND-1000; NanoDrop Technologies USA). The total RNA was reverse transcribed into first-strand cDNA using the RevertAid First-Strand cDNA Synthesis Kit (MBI Fermentas, USA). The open reading frame (ORF) of *B. mori E(spl)m4* was amplified by PCR using the cDNA as a template and the primers 5′-CGGGATCCATGTCTACGTACGC-3′ and 5′- CGGAATTCTCAAGCTTGCACCC -3′, which contained *Bam*H I and *Eco*R I (MBI, Canada) restriction sites, respectively.

The PCR was performed using a Taq DNA Polymerase Kit (Promega, USA) and the following reaction conditions: an initial denaturation at 94°C for 5 minutes, followed by 30 cycles of denaturation at 94°C for 40 seconds, annealing at 61°C for 40 seconds, and extension at 72°C for 45 seconds, with a final extension step at 72°C for 10 minutes. The resulting PCR product was purified using the PCR Rapid Purification Kit (BioDev-Tech, China). After digestion with *Bam*H I and *Eco*R I, the purified product was subcloned into the expression vector pET-28a (Amersham Biosciences, USA) using T4 DNA ligase (Promega, USA) and transformed into *E. coli* TG1 cells (maintained in our laboratory) for screening purposes. A positive colony was identified by PCR and double digestion of the purified plasmid followed by electrophoresis on a 1% agarose gel. The construct was subsequently verified by sequencing with the BigDye Terminator method on an ABI PRISM 3130XL/A automated sequencer (Applied Biosystems, USA).

The recombinant plasmid pET-28a-*BmE(spl)m4* was transformed into *E. coli* cells (strain BL21(DE3)), and expression of the His-tagged fusion protein was induced with 1 mM isopropylthiogalactoside (IPTG) at 37°C for 5 hours. After the culture medium had been removed, the bacterial pellets were frozen and thawed three times and then resuspended in lysis buffer (20 mM Tris, 200 mM NaCl, pH 8.0). The bacteria were then disrupted with an Ultrasonic Crasher Φ2 cell disruptor (Ningbo Scientz, China) for 40 minutes on ice. The lysates were centrifuged at 12 000 g for 20 minutes at 4°C to remove cell debris and other particles. The precipitated phase was collected and washed with inclusion washing buffer I (20 mM Tris, 1% Triton-X100, pH 8.0), followed by inclusion washing buffer II (20 mM Tris, 3 mol/L Urea, 50 *μ*M ZnCl, pH 8.0), three times each, and lysis buffer once. It was then dissolved in phosphate-buffered saline (PBS) that contained 8 mol/L urea overnight. The resulting solution was centrifuged at 14 000 g for 30 minutes at 4°C to remove cell debris. The supernatant was collected and filtered through a 0.45 *μ*m filter (Millipore, USA) and then purified using Ni Sepharose 6 Fast Flow (GE Healthcare, USA) according to the manufacturer's instructions. Purified rBmEm4 was analyzed by SDS-PAGE and western blotting with an antibody against the His6-tag (diluted 1/1000; Santa Cruz, USA) and then identified with a 4700 MALDI-TOF/TOF mass spectrometer (ABI, USA). The recombinant protein was then injected into a New Zealand rabbit to generate a polyclonal antibody [26]. The antiserum was purified using a HiTrap Protein A HP column (GE Lifesciences, USA) following the manufacturer's instructions. An ELISA was used to determine the titer of the polyclonal antibody. The specificity of the polyclonal antibody was determined by Western blotting analysis: the purified polyclonal antibody was diluted 1 : 500 and incubated with the immunoblot at room temperature for 2 hours; the goat antirabbit secondary antibody (LI-COR, USA) was diluted 1 : 1000.

### 2.4. Analysis of BmEm4 Expression by Semiquantitative RT-PCR

Total RNAs from various silkworm developmental stages and from tissues from day-3 fifth instar larvae (trachea, head, fatty body, gut, testis, Malpighian tubule, epidermis, ovary, and silk gland) were isolated using Trizol reagent (Invitrogen) according to the manufacturer's instructions. Contaminating gDNA was removed by treatment with DNase I (TaKaRa). The purity of the extracted RNA was determined by UV spectrophotometry (NanoDrop ND-1000). Equivalent amounts of total RNA were reverse transcribed into first-strand cDNA using the RevertAid First-Strand cDNA Synthesis Kit (MBI). The primers used for the real-time quantitative reverse transcription PCR (qRT-PCR) were designed using the Primerselect program from the Primer 5.0 software (PREMIER Biosoft International, USA). A pair of primers was also designed to amplify 18S ribosomal RNA (rRNA), which was used as an internal control. The sequences of the primers used for the real-time qRT-PCR were as follows:

– (18S rRNA): Forward primer, 5′-CGATCCGCCGACGTTACTACA-3′;
Reverse primer, 5′-GTCCGGGCCTGGTGAGATTT-3′.
– (*BmEm4*): Forward primer, 5′-AGATGAAACCTGTGACCTTGTGCC-3′;
Reverse primer, 5′-CTTGGAGCACTGGATGTTGTTGTTC-3′.

The real-time qRT-PCR was performed in an ABI Prism 7300 Sequence Detection System (Applied Biosystems) using a SYBR Green Real-time PCR Master Mix-Plus- (QPK-212) Kit (Toyobo, Japan). The reaction mixtures (10 *μ*L) contained 5 *μ*L of 2 × SYBR-Green Reaction mix, 0.2 *μ*L of 10 *μ*M forward and reverse primers, respectively, 0.5 *μ*L of cDNA, and 4.1 *μ*L of diethylpyrocarbonate (DEPC)-treated water. The reaction conditions used for the real-time qRT-PCR were pre-denaturation at 95°C for 1 minute, followed by 40 cycles of 95°C for 15 seconds, 60°C for 15 seconds, and 72°C for 45 seconds. The dissociation curve was determined using the following conditions: 95°C for 15 seconds, 60°C for 30 seconds, and 95°C for 15 seconds, to check for the presence of nonspecific dsDNA hybrids, such as primer-dimers. Each reaction was performed in triplicate in a 96-well plate along with the 18S rRNA control. The expression levels of the target genes were normalized against the expression levels of the 18S rRNA gene. Relative expression levels were calculated using the 2^−ΔΔCT^ method for RNA quantification where ΔΔCT = (CT, target gene–CT,18S rRNA)_different stages or tissues_−(CT, target gene−CT, 18S rRNA)_maximum_ [27].

### 2.5. Analysis of BmEm4 Expression by Western Blotting

To prepare protein extracts, silkworms at different developmental stages or different tissues were frozen in liquid nitrogen, ground into a fine powder, and treated with protein extraction lysate buffer (50 mM Tris pH 8.0, 0.15 M NaCl, 5 mM EDTA, 0.5% NP-40, 1 mM dithiothreitol, 5 g/L sodium deoxycholate, 100 mg/L PMSF, 5 *μ*g/mL Aprotin). After 30 minutes on ice, the homogenates were centrifuged at 12 000 g for 15 minutes at 4°C. The proteins in the supernatant were separated by SDS-PAGE on a 12% gel and then electroblotted onto polyvinylidene fluoride (PVDF) membrane (Millipore, USA). The protein concentration in the samples was determined using the BCA Protein Assay kit (Pierce, USA) following the manufacturer's instructions and equalized prior to SDS-PAGE. For the different developmental stages, 127 *μ*g of protein were loaded in each lane, whereas 146.4 *μ*g were loaded for the different tissues from fifth instar larva. Following blocking and incubation with the purified anti-BmEm4 antibody, the membranes were washed and incubated with donkey antirabbit IgG (LI-COR). Finally, the membranes were washed three times with PBS plus 0.1% Tween-20, twice with PBS without Tween-20, and subsequently scanned using an Odyssey Infrared Imaging System (LI-COR) at 480 nm. The sizes of the proteins were determined by comparison with a prestained protein molecular weight marker (Beyotime, China).

### 2.6. Immunohistochemistry

Fifth instar larvae, which had just started spinning the cocoon, and pupae, at three days after pupation, were frozen in liquid nitrogen and embedded in OCT compound at temperatures below −20°C. Sections (10 *μ*m thick) were cut and attached to microscope slides. The sections were then treated with cold acetone, washed three times in PBS for 15 minutes, treated with 3% bovine serum albumin (BSA) in PBS for 2 hours, and incubated for 2 hours with purified anti-BmEm4 IgG (1 : 100). After three washes in PBST (PBS plus 0.1% Tween-20), the sections were incubated with antirabbit IgG conjugated to Alexa Fluor 405 (diluted 1 : 500; Molecular Probes, USA) for 1 hour, washed three times in PBST and twice in PBS, and then observed by fluorescence microscopy at 405 nm. As a control, sections were incubated with antibody against BmEm4 that had been preabsorbed with excess H6-tagged BmEm4 fusion protein.

### 2.7. Subcellular Localization of BmEm4

Cells were cultured overnight on a covered dish that was used specifically for confocal microscopy. After the culture medium had been removed, the cells were washed three times for 5 minutes in PBS and fixed in 4% polyformaldehyde in PBS (pH 7.4) at room temperature for 15 minutes. The fixed cells were blocked in 3% BSA in PBS at room temperature for 2 hours and then washed three times for 10 minutes in PBST. The cells were then incubated with the anti-BmEm4 polyclonal antibody (diluted 1 : 500 in blocking buffer) at 37°C for 2 hours; cells were incubated simultaneously with control serum. The control serum was obtained from the rabbit before immunization with the antigen. After three 10 minutes washes in PBST, the cells were incubated with Cy3-labeled goat antirabbit IgG (diluted 1 : 1000; Promega) at 37°C for 1 hour and then again washed three times for 10 minutes in PBST. The cells were then incubated with 4-6-diamidino-2-phenylindole (1 g/mL in PBS) at room temperature for 10 minutes. After the cells were washed once with PBST, they were examined under a Nikon Eclipse TE2000-E Confocal Microscope (Nikon, Japan). Images were analyzed using EZ-C1 software (Nikon, Japan). Cy3-labeled goat antirabbit IgG emits red fluorescence when stimulated with light of wavelength 550 nm, and DAPI-stained nuclei emit blue fluorescence when stimulated with light of wavelength 353 nm.

## 3. Results

### 3.1. Bioinformatics Analysis

**Figure 1:**
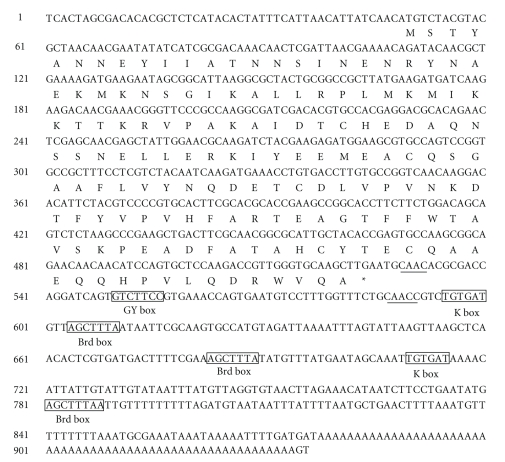
Nucleotide and deduced amino acid sequence for* Bombyx mori E(spl)m4* (*BmEm4*). Conserved GY-box (GTCTTCC), K-box (TGTGAT), and Brd-box (AGCTTTA) motifs were found in the 3′ UTR (defined in Leviten et al. [19]; Lai et al. [20]) and are marked by boxes. The sequence also contains two CAAC motifs in the 3′ UTR, which are underlined.

**Figure 2:**
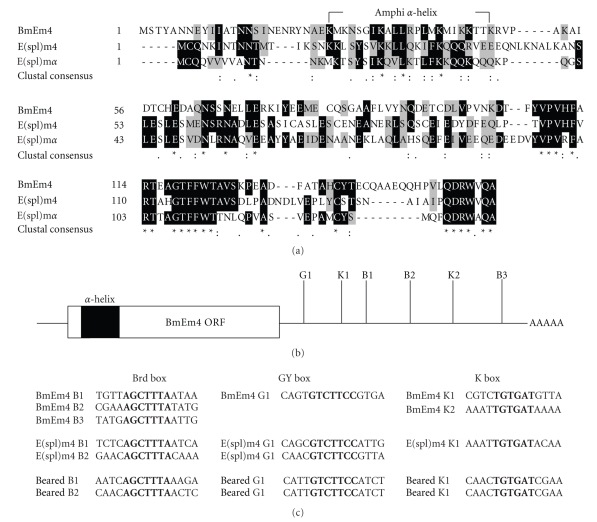
Sequence alignment illustrating the conserved motifs of the *BmEm4* gene. (a) Sequence comparisons of *Bombyx mori E(spl)m4* (accession number FJ436408), *Drosophila melanogaster E(spl)m4* (accession number X16551), and *E(spl)* *m* *α* (accession number AJ011140). Amino acid residues that are identical between the BmEm4, E(spl)m4, and E(spl)m*α* proteins are shown by dark boxes and similar amino acids are lightly shaded. A star under the sequences indicates identical amino acids; two points indicate a high degree of conservation; one point indicates a low degree of conservation. The basic amphipathic *α*-helical regions of each protein as predicted by protean software are bracketed (amino acids 26–44 for BmEm4). The other two sequences that are conserved in the Brd family (VPVHFARTXXGTFFWT and DRW(A/V)QA) are found at the C-termini of the proteins (in shadow). (b) The locations of the conserved motifs including the GY, K, and Brd boxes (indicated by G, K, and B) in the 3′ UTR of *BmEm4 *are shown. (c) Alignment of the Brd-box (AGCTTTA), GY-box (GTCTTCC), and K-box (TGTGAT) motifs found in the 3′ UTRs of *BmEm4*, *E(spl) m4, Beared* genes (defined in Leviten et al. [19]), and *Barbu* (defined in Zaffran and Frasch [39]). Each sequence motif in a given gene is numbered in the order of its position 5′ to 3′ in the 3′ UTR. The core of each box is shown in bold letters.

The nucleotide sequence of the *B. mori E(spl)m4* gene (accession number FJ436408) and the deduced amino acid sequence that it encodes are shown in Figure 1. Sequence analysis revealed that the ORF of *BmEm4* was 477 bp in length and encoded a putative protein of 158 amino acids, with a predicted molecular mass of 18.01 kD. When submitted to BLAST, the deduced amino acid sequence displayed significant homology to two *Drosophila* proteins, namely, m4 and m*α* of the E(spl)-C [9, 13]. These small proteins are predicted to contain an *α*-helix with basic amphipathic structure in their N-terminal region, and this structure appears also to be present in BmEm4 [13, 19]. In addition, two other amino acid sequences that are conserved in the Brd family (VPVHFARTXXGTFFWT and DRW(A/V)QA) are found in the C-termini of all three proteins (Figure 2(a)) [12]. Domain structure analysis revealed that the 3′ UTR of *BmEm4* contained three Brd boxes (AGCTTTA), one GY box (GTCTTCC), two K boxes (TGTGAT), and two CAAC motifs (Figure 2(b)). All these motifs are found in the 3′ UTRs of members of the *Brd* gene family (Figure 2(c)).

### 3.2. Heterologous Expression of BmEm4 and Preparation of a Polyclonal Antibody

The *B. mori E(spl)m4* gene was identified initially as a potential miRNA target gene in our laboratory [25]. In an effort to gain a more complete understanding of the structure and function of this gene, we amplified the cDNA for BmEm4 using specific RT-PCR primers and total RNA extracted from silkworm pupa. A polyclonal antibody against BmEm4 was generated by injecting a New Zealand rabbit with purified recombinant rBmEm4 protein and purified by HiTrap Protein A HP column chromatography. The titer of the anti-BmEm4 polyclonal antibody, as determined by ELISA, was 1 : 32 000, and western blot analysis indicated that the antibody reacted specifically with BmEm4-H6 (data not shown).

### 3.3. Expression Analysis of BmEm4

**Figure 3:**
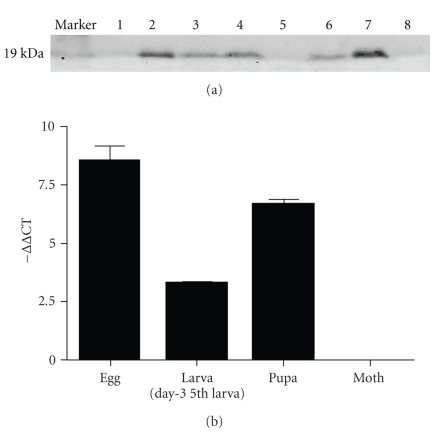
Levels of transcription and translation of *BmEm4* in different developmental stages. BmEm4 expression was analyzed by Western blotting (a) and real-time qRT-PCR (b). The relative level of transcription was calculated using 2^−ΔΔCT^ (1, egg; 2, first instar larva; 3, second instar larva; 4, third instar larva; 5, fourth instar larva; 6, fifth intar larva; 7, pupa; 8, moth). The results shown are representative of three independent experiments.

**Figure 4:**
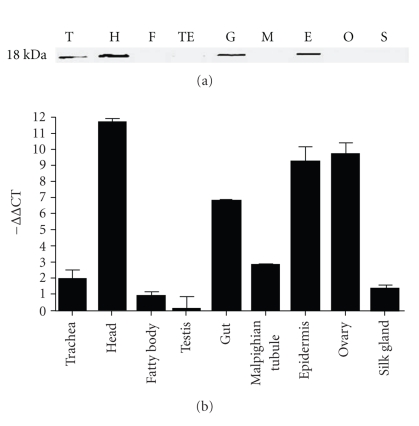
Levels of transcription and translation of *BmEm4* in different tissues from day-3 fifth instar larvae. (a) Western blot analysis. Protein extracts from the trachea (T), head (H), fatty body (F), testis (TE), gut (G), Malpighian tubule (M), epidermis (E), ovary (O), and silk gland (S) of fifth instar larvae were subjected to immunoblotting. (b) Real-time qRT-PCR analysis. The relative expression level was calculated using 2^−ΔΔCT^. Three independent experiments were performed.

We employed real-time qRT-PCR to quantify *BmEm4* messenger RNA (mRNA) levels in various developmental stages of silkworm and in fifth instar larval tissues. Our results indicated that the *B. mori E(spl)m4* gene was highly expressed in fifth instar larvae and pupae. In day-3 fifth instar larvae, it was expressed abundantly in the head, ovary, epidermis, and testis. We also performed western blot analyses to determine the levels of* B. mori* E(spl)m4 protein in eggs, larvae of each instar, pupae, moths, and different tissues from day-3 5th instar larvae. The results showed that BmEm4 expression was barely detectable in eggs. It began to emerge in first instar, second instar, and third instar larvae, but it decreased significantly in fourth instar larvae. The expression increased again in fifth instar larvae, reached a peak in pupae, and finally disappeared in the moth. When the mRNA and protein levels were compared, it could be seen that the level of protein did not correlate with the level of mRNA during some developmental stages (Figure 3). The egg contained the highest level of mRNA, but the highest protein level was observed in the pupa and the protein was hardly detectable in the egg. The results indicate that the translation of *BmEm4* mRNA in the egg may be inhibited. The expression level of BmEm4 protein in different tissues from the fifth instar larvae was highest in the head, followed by the gut and epidermis; the protein was barely detectable in the trachea, fatty body, testis, Malpighian tubule, ovary, and silk gland. The results of the real-time qRT-PCR showed that *BmEm4* was highly transcribed in the head, ovary, epidermis, and gut (Figure 4).

### 3.4. Immunohistochemistry Analysis

**Figure 5:**
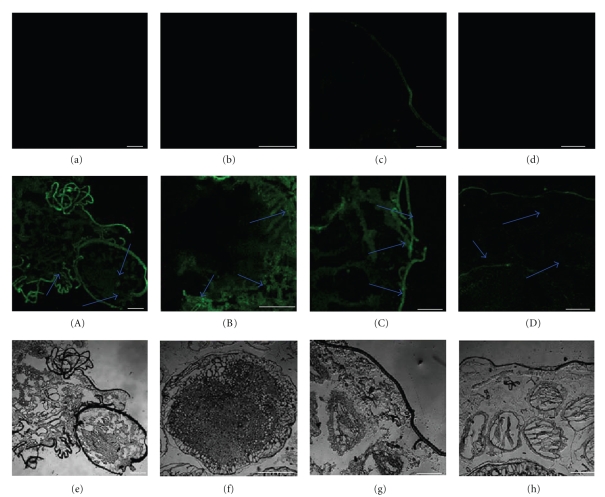
Immunohistochemical staining of BmEm4 in fiftth instar larvae that had just started to spin. (a)–(d) Negative controls for (A)–(D), respectively. (a) (A) head; (b) (B) silk gland; (c) (C) epidermis; (d) (D) ovary. The arrows show immunopositive signals for BmEm4. (e)–(h) are the bright field. The scale bars correspond to 400 *μ*m.

**Figure 6:**
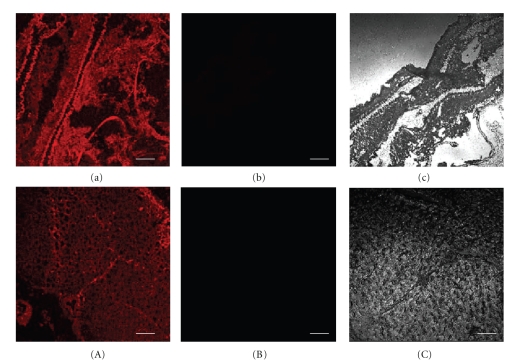
Immunohistochemical staining of BmEm4 in day-3 pupae. (b) and (B) correspond to negative controls for (a) and (A) respectively. (c) and (C) are photographed under the light microscope. (a), (b), (c) show the fat body and body wall; (A), (B), (C) show the testis. The scale bars correspond to 400 *μ*m.

To determine the tissue-specific localization of BmEm4, frozen sections were prepared from fresh fifth instar larvae (which had just started to spin the cocoon) that had been quick-frozen in liquid nitrogen. The sections were subjected to immunohistochemistry with the purified anti-BmEm4 antibody. We found that BmEm4 was expressed in the head, silk gland, and ovary of fifth instar larvae (Figure 5). The epidermis is autofluorescent. Using western blotting, we also determined that the highest level of BmEm4 expression during the pupal stage occurred on day 3. Therefore, we chose to study day-3 pupae in our immunohistochemical analyses. The results revealed that BmEm4 is highly expressed in the skin and subcutaneous fat of the pupa (Figure 6).

### 3.5. Subcellular Localization of BmEm4

**Figure 7:**
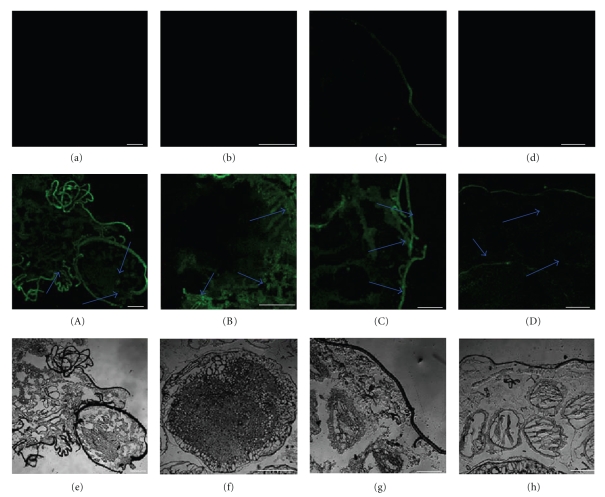
Subcellular localization of BmEm4 with Cy3-labeled goat antirabbit IgG and DAPI. (a)–(d), (e)–(h), (i)–(l) correspond to three individual experimental groups, which were stained with the anti-BmEm4 polyclonal antibody. (a) and (e) the subcellular localization of BmEm4 as indicated by the Cy3-labeled secondary antibody; (b) and (f) DAPI staining; (c) and (g) show the previous two images merged together; (i)–(l) negative control group, using control rabbit serum; (l), signal obtained with Cy3-labeled secondary antibody; (j) DAPI staining; (k), merged images from (i) and (j); (d), (h), (l) are bright field images.

The treated cells were examined under a Nikon ECLIPSE TE2000-E confocal microscope, and images were analyzed using EZ-C1 software. Our results indicated that BmEm4 was located in both the cytoplasm and nucleus but was found primarily in the cytoplasm (Figure 7).

## 4. Discussion

### 4.1. Bioinformation Analysis of BmEm4

The *E(spl)m4* gene was identified as a miRNA target gene in *Drosophila* [28]. We obtained the ortholog of the *E(spl)m4* gene from *B. mori* (*BmEm4*). The amino acid sequence of *BmEm4* showed homology to that of two other *Brd* family genes *E(spl)m4* and *m* *α*. Although the degree of identity between the full-length amino acid sequences of these genes is low, we identified a putative basic amphipathic *α*-helical domain that is largely conserved between all three proteins. Basic amphipathic *α*-helixes are associated with a variety of functions, which include protein-protein interaction (e.g., binding of calmodulin) and interaction with lipid membranes [29]. Furthermore, the most prominent and highly conserved sequence features (such as the Brd box, GY box, K box, and CAAC motif) were found in all three genes. BmEm4 shares a common sequence and secondary structure characteristics with members of the Brd protein family. We have shown that it is a novel gene in silkworm that is related to *E(spl) m4* and *m* *α*.

### 4.2. Analysis of the Expression and Regulation of BmEm4

It is well known that the invertebrate head contains a highly functional nervous system. Our analysis of the levels of *BmEm4* mRNA and protein in various tissues from the fifth instar larva showed that the expression of both mRNA and protein was much higher in the head than in other tissues (Figure 4). This suggests that both the mRNA and protein products of *E(spl)m4* could be involved in the regulation of neurogenesis [17]. In Figure 5(a), the silk-like fluorescent signal corresponds to the epidermis of the head and neck, and the positive signals indicated by the arrows are the brain and subpharyngeal nerve. In addition, BmEm4 was expressed in the epidermis, which contains an abundance of neurons to respond to external stimulation. Our observations provide evidence that both the mRNA and protein products of *BmEm4* have the capacity to be involved separately in the regulation of neurogenesis, [12]. Furthermore, BmEm4 is also expressed in the trachea and gut. This suggests that BmEm4 may be involved in the development of the respiratory and digestive systems of the silkworm.

The main reason that we chose to study fifth instar larvae, the developmental stage at which the silkworm is about to begin spinning its cocoon, is that, at this stage, the silkworm does not consume mulberry leaves, which might affect results obtained from the intestinal tract. In addition, fifth instar larvae have a completely developed apparatus, a large silk gland, and definitive genitalia. In fifth instar larvae that had just started spinning the cocoon, immunoblot analyses revealed that BmEm4 was expressed in the silk gland. Therefore, we presumed that BmEm4 is involved in the growth and development of silk glands and the production of silk sericins.

Immunohistochemical analyses of day-3 pupae revealed that BmEm4 is highly expressed in the epidermis and subcutaneous fat. Moreover, western blot analysis of day-3 fifth instar larvae revealed that BmEm4 is hardly detectable in the ovary, However, in the “just spinning” stage, it is expressed slightly in the ovary, and immunohistochemical analyses show that it is highly expressed in the ovary of day-3 pupa. Therefore, the expression of BmEm4 increased significantly as the ovary developed and matured, which suggests that BmEm4 plays a positive role in the development of the ovary. It is known that Notch signaling plays a fundamental role in the correct specification of cell fates in multicellular animals [1–3]. In the silkworm, *B. mori*, the changes in morphology and bodily activities that are necessary for the fertilized ovum to develop into a fully-fledged moth occur within 40 days. In the pupal stage, in particular, histolysis and histogenesis occur intensively and simultaneously. Consequently, the fact that the highest levels of expression of BmEm4 occur in the pupal stage suggests that it may be involved in the Notch signaling pathway.

**Figure 8:**
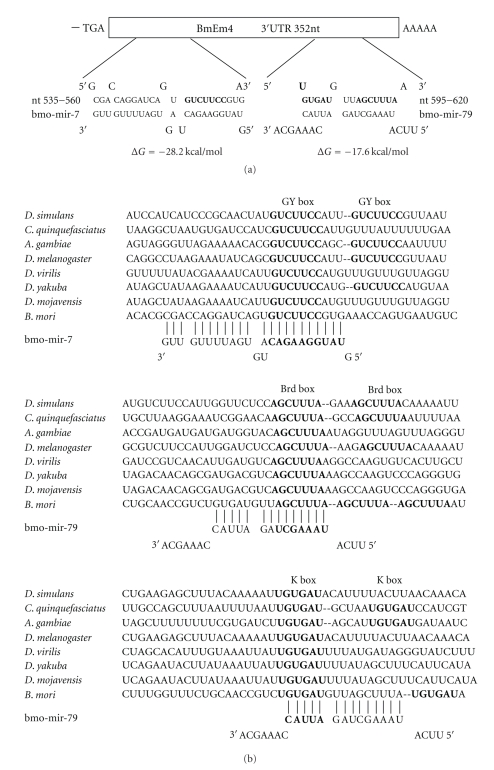
The *BmEm4 *3′ UTR contains a *bmo-mir-7* target site at nt 535–560 and a *bmo-mir-79 *target site at nt 595–620. In addition, different classes of miRNA binding site (GY boxes, K boxes, and Brd boxes) are highly conserved in the 3′ UTR of the *m4* ortholog from eight different species. (a) The locations of the putative *bmo-mir-7* and *bmo-mir-79* sites are shown. The target site is shown in bold, and unpaired bases are shown above and below the duplex. (b) Comparison of the *bmo-mir-7* and *bmo-mir-79* seed sequences and their targets in eight species.

miRNAs are single-stranded noncoding RNAs that range in length from 19 nucleotides (nt) to 25 nt and modulate gene expression in both plants and animals. A large number of miRNAs are conserved evolutionarily across species boundaries [30]. A series of genetic and bioinformatic studies over the past decade have suggested that the Notch signaling pathway in *Drosophila* is regulated by miRNAs [28, 31, 32]**.** Subsequently, GY-box, Brd-box, and K-box motifs have been shown to be perfectly complementary to the 5′ ends of various *Drosophila* miRNAs, which suggests a direct regulatory relationship between miRNAs and these motifs [33]. Indeed, two E(spl)-C genes that contain GY boxes have been validated as targets of *miR-7* [34]. As shown in Figure 8(b), we found that three different classes of miRNA binding site (GY boxes, Brd boxes, and K boxes) are highly conserved in the 3′ UTRs of m4 orthologs from eight different species (including *BmEm4*). This implies that *BmEm4* might also be regulated by miRNAs. Sequence analyses have shown that some mature miRNAs are conserved between *B. mori *and *D. melanogaster* [35, 36]. Therefore, miRNAs that target the GY, Brd, and K boxes may also exist in *B. mori* and regulate the *BmEm4* gene; this would be consistent with the observation that most Notch target genes in *Drosophila* (including *E(spl)m4*) are negatively regulated by miRNAs [28]. Previously, we identified 46 *B. mori* miRNAs, two of which were predicted to regulate the *BmEm4* gene via its 3′ UTR [25]. The minimum free energy for hybridization as predicted by mFold analysis is −28.2 kcal/mol between *BmEm4* and *bmo-mir-7* and −17.6 kcal/mol between *BmEm4* and *bmo-mir-79* (Figure 8(a)). From Figure 3, we can clearly observe that *BmEm4* mRNA was highly transcribed in both eggs and pupae, whereas the results of western blotting indicated that BmEm4 protein was highly expressed in pupae but was hardly detectable in eggs. According to these findings, it appears that *BmEm4* may be modulated by miRNAs. However, the expression of mRNAs can also be regulated by other mechanisms, such as RNA binding proteins that act as translational regulators [37, 38]. Alternatively, the lack of correlation between the levels of mRNA and protein may be explained by the stability of the BmEm4 protein.

### 4.3. Analysis of the Subcellular Localization of BmEm4

We found that, in the Bm5 cell line, BmEm4 was localized to both the cytoplasm and nucleus but was found primarily in the cytoplasm. The Notch-activated transcription factor SuH is also found in both the nucleus and cytoplasm [39], and previous data support an interaction between E(spl)m4 and SuH in vivo [6, 15, 16]. It is known that the majority of proteins that play a role in the nucleus are normally located in the cytoplasm and only translocate to the nucleus when activated to perform a specific function. The nucleus contains low levels of Brd family proteins, which are therefore difficult to detect by antibody staining [40]. This situation could be similar to that for the intracellular domain of Notch, which is also barely detectable in the nuclei of wild type embryos by antibody staining, although more sophisticated techniques have demonstrated that it does function within the nucleus [40, 41].

In summary, we have identified a *B*. *mori E(spl)m4* gene that is expressed in a stage- and tissue-specific manner and propose that it may involved in the regulation of neurogenesis. We have observed differences between the mRNA and protein levels for BmEm4 during development, which raises the possibility of posttranscriptional regulation of the *BmEm4* mRNA. Consistent with this idea is the finding that the *BmEm4 *3′ UTR contains two putative binding sites for miRNAs. Moreover, immunostaining indicated that BmEm4 can be found in both the nucleus and cytoplasm but is located primarily in the cytoplasm. However, the actual physiological function and regulatory mechanism of *BmEm4* in the silkworm requires further investigation, and this is the next aim of our research.

## Abbreviations

3′ UTR: 3′ untranslated region
cDNA: DNA complementary to RNA
ELISA: Enzyme linked immunosorbent assay
E(spl)-C: Enhancer of split complex
H6-tag: His_6_tag
IPTG: Isopropyl-*β*-D-thiogalactopyranoside
KDa: Kilodaltons
miRNA: MicroRNA
mRNA: Messenger RNA
nt: Nucleotide(s)
ORF: Open reading frame
PMSF: Phenylmethanesulfonyl fluoride
qrt-PCR: Quantitative reverse transcription polymerase chain reaction
RBPs: RNA-binding proteins
SDS: Sodium dodecyl sulfate.
